# Clinical and demographic factors affecting disease severity in patients with multiple sclerosis

**Published:** 2013

**Authors:** Somayeh Baghizadeh, Mohammad Ali Sahraian, Nahid Beladimoghadam

**Affiliations:** 1 Neurologist, Bouali Hospital, Qazvin University of Medical Sciences, Qazvin, Iran; 2 Associate Professor, Department of Neurology, Sina Hospital, Tehran University of Medical Sciences, Tehran, Iran; 3 Assistant Professor, Department of Neurology, Imam Hossein Hospital, Shahid Beheshti University of Medical Sciences, Tehran, Iran

**Keywords:** Multiple Sclerosis, Severity, Demographic, Multiple Sclerosis Severity Score (MSSS)

## Abstract

**Background:**

The clinical course of multiple sclerosis (MS) evolves over many years. Its prognosis is highly variable among affected individuals, i.e. while some suffer from early severe disabilities, others remain ambulatory and functional for many years. We used Multiple Sclerosis Severity Score (MSSS) and the new classification for MS severity Herbert et al. introduced in 2006 according to MSSS, to investigate some clinical and demographic factors as potential indicators of disease severity in in MS.

**Methods:**

During a six-month period, patients with definite MS according to the revised McDonald's criteria who referred to three neurology and MS clinics in Tehran (Iran) were included in the study. All patients were interviewed and examined by a neurology resident who had been trained for employing the Expanded Disability Status Scale (EDSS). For each patient, MSSS was determined by using EDSS and disease duration.

**Results:**

Overall, 338 (266 female and 72 male) patients were enrolled. Among demographic features, gender, younger age at onset, positive family history, and parental consanguinity were not associated with disease severity. Education was weakly associated with disease severity. Among clinical factors, presenting symptoms such as poly-symptomatic attacks, walking difficulty, and upper and lower extremity dysfunction were associated with more disability while presentation with optic neuritis had better prognosis. Complete recovery after the first attack, longer interval between the first and second attacks, lower number of symptoms at presentation, shorter duration of attacks, and relapsing-remitting course were associated with less disability and better prognosis. These results were noticed in ordinal logistic regression. However when multiple logistic regression was performed, the strongest determinant of disease severity was disease course with odds ratio (OR) = 49.12 for secondary progressive course and OR = 53.25 for primary progressive (± relapse) course. Walking difficulty as the presenting symptom had a borderline association with disease severity (OR = 2.31; P = 0.055). Increased number of onset symptoms was associated (but not significantly) with more severe disease.

**Conclusion:**

Early prediction of disease severity by demographic and clinical features is currently impossible. We need to determine stronger predictors, possibly a combination of demographic, clinical, biomarkers, and imaging findings.

## Introduction

The clinical course of multiple sclerosis (MS) evolves over many years. The disease has a highly variable prognosis causing early severe disabilities in some patients but leaving others ambulatory and functional for many years.^1–3^ Predictors of this variable course have long been investigated and can be helpful for many reasons, e.g. to predict long-term course of the disease at individual level, to help in initiating disease-modifying drugs and treatment selection, to give some insight about the pathogenesis of MS, and to identify modifiable prognostic factors. However, there are no established paraclinical methods to predict disease severity. Possible clinical and demographic predictors have been largely assessed but there is not agreement on all of them. To determine possible predictive factors, one needs a reliable scoring system to evaluate disability and disease severity. Unfortunately, the currently available tools have major disadvantages. The Expanded Disability Status Scale (EDSS) neither reflects disease activity at one particular point of time, nor considers some neurological disabilities such as fatigue, cognitive dysfunction, or pain common in patients with MS. Annual relapse rate, frequently used as a measure of disease activity, does not necessarily translate into disability. On the other hand, there is no consensus on radiological features that may serve as surrogate markers of disease activity or patient disability. EDSS, annual relapse rate, or radiological features do not take into account the important aspect of disease duration, which is a major factor in accumulation of central nervous system damage over time and functional disability.

MS Severity Score (MSSS) is a newly introduced tool. Based on databases in 10 European countries and Australia, the authors collected two critical elements of information, i.e. disease duration in years and EDSS score, from 9,892 patients. The algorithm relates a patient's EDSS score to the distribution of disability in patients with the same disease duration. Thus, similar relatively high MSSS numbers will be assigned to patients who develop moderate disability over a short period of time or severe disability over a moderate period of time.^4^ Most studies on disease severity were confined to chronic MS with different definitions (EDSS < 2 or 3 after 10 or 20 years) and thus required the follow-up of patients for a long period to conclude chronicity. While most previous studies lacked severity subgroups with a specific definition, Herbert recently introduced eight subgroups of disease severity according to MSSS.^5, 6^

We used MSSS and Herbert's classification of MS severity according to MSSS to study a number of clinical and demographic factors as possible indicators of disease severity in some referral MS clinics in Iran.

## Materials and Methods

**Figure 1:**
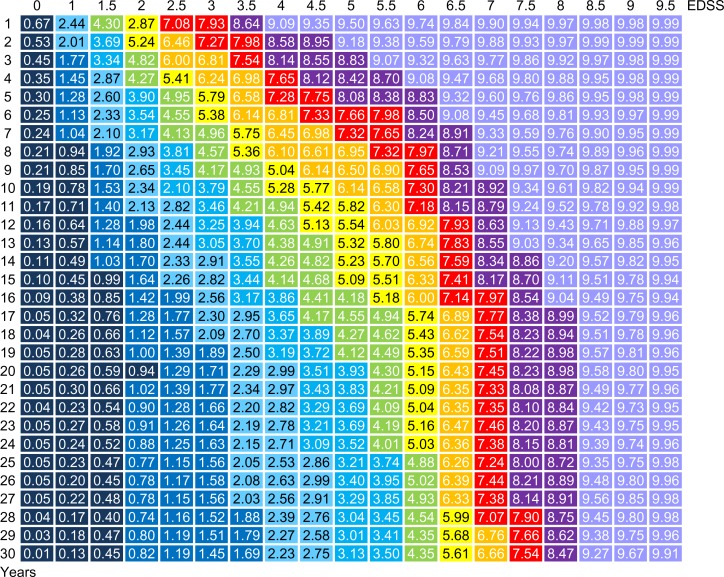
Matrixes of Expanded Disability Status Scale (EDSS) score as a function of disease duration showing the distribution of Multiple Sclerosis Severity Score (MSSS) deciles

During October 2010-March 2011, all patients with definite MS (according to the Revised McDonald Criteria, 2005) attending three neurology and MS clinics in Tehran (Iran) were included in this study. Patients with relapse in the past three months and those with disabilities due to reasons other than MS which could confound EDSS score determination were excluded. All patients were interviewed and examined by a neurology resident educated for EDSS score determination. Data was recorded in appropriate data sheets. MSSS of each patient was calculated using EDSS and disease duration (Figure 1).

Patients were categorized in four subgroups (Table 1) and the following variables were studied: gender, age at disease onset, disease duration, education, positive family history for MS, parental consanguinity, disease course, number of symptoms at onset, presenting symptom, recovery from first attack, and interval between the first and second attacks.

**Table 1:** Disease severity subgroups according to the Multiple Sclerosis Severity Score (MSSS)^5, 6^

	MSSS
Benign	≤ 0.45
Mild-moderate	0.46-5.00
Advanced-accelerated	5.00-8.23
Aggressive-malignant	≥ 8.24

All analyses were performed using SPSS for Windows 17.0 (SPSS Inc., Chicago, IL, USA). Descriptive variables were presented as mean, standard deviation, frequency, mode, and percent. Associations of MSSS with clinical and demographic factors were examined by Mann-Whitney and Kruskal-Wallis tests and Spearman Correlation. An ordinal logistic regression was performed and odds ratio (OR) for getting worse conditions (more severe disease) was calculated for each variable. Finally, a multiple ordinal logistic regression analysis with backward selection was used to select the set of covariates that were independently associated with the outcome. P-values of less than 0.05 on two-tailed tests were considered as statistically significant.

## Results

A total of 338 (266 female, 72 male) patients enrolled in our study. Female to male ratio was 3.69.The mean age of the patients and mean age at disease onset were 34.0 ± 10.0 and 24.6 ± 8.7 years, respectively.

### Results of univariate analysis

Among demographic features, gender, younger age at onset, positive family history, and parental consanguinity were not associated with disease severity. However, a weak relationship was detected between education and disease severity (Table 2).

**Table 2:** Associations of demographic factors and disease severity

		Total	Chronic	Mild-moderate	Advanced-accelerated	Aggressive- malignant	P
Gender	Male	72	8 (11.1)	30 (41.7)	16 (22.2)	18 (25.0)	0.271†
	Female	266	31 (11.7)	121 (45.5)	75 (28.2)	39 (14.7)	
Education (years)	Illiterate	0	0 (0)	0 (0)	0 (0)	0 (0)	< 0.001*
	< 5	19	0 (0)	3 (15.8)	8 (42.1)	8 (42.1)	
	5-12	136	11 (8.1)	55 (40.4)	39 (28.7)	31 (22.8)	
	12-16	94	11 (11.7)	53 (56.4)	19 (20.2)	11 (11.7)	
	> 16	18	2 (11.1)	10 (55.6)	3 (16.7)	3 (16.7)	
Parental consanguinity	No	183	18 (9.8)	86 (47.0)	44 (24.0)	35 (19.1)	0.745†
	Yes	41	3 (7.3)	19 (46.3)	12 (29.3)	7 (17.1)	
Positive family history	No	178	18 (10.1)	82 (46.1)	45 (25.3)	33 (18.5)	0.804^‡^
	Yes	46	3 (6.5)	23 (50.0)	11 (23.9)	9 (19.6)	

† Based on Mann-Whitney test

* Based on Spearman correlation

Among clinical factors, presenting symptoms such as difficulty in walking, polysymptomatic attacks, and upper and lower extremity dysfunction were associated with greater disability. In contrast, presenting optic neuritis had better prognosis (Table 3).

**Table 3:** Associations of the presenting symptoms and disease severity

	Total	Chronic	Mild-moderate	Advanced-accelerated	Aggressive-malignant	P*
Polysymptomatic onset	169	10 (25.6)	71 (47.0)	50 (54.9)	38 (66.7)	< 0.001
Difficulty in walking	96	3 (7.7)	29 (19.2)	35 (38.9)	29 (50.9)	< 0.001
Lower extremity dysfunction	71	3 (7.7)	16 (10.6)	31 (34.1)	21 (36.8)	< 0.001
Upper extremity dysfunction	45	2 (5.1)	17 (11.3)	16 (17.6)	10 (17.5)	0.030
Reduced visual acuity (optic neuritis)	114	13 (33.3)	61 (40.4)	28 (30.8)	12 (21.1)	0.031
Sexual dysfunction	1	0 (0)	0 (0)	0 (0)	1 (1.8)	0.172
Bladder/bowel dysfunction	21	2 (5.1)	6 (4.0)	9 (9.9)	4 (7.0)	0.190
Fatigue	31	1 (2.6)	15 (9.9)	8 (8.8)	7 (12.3)	0.261
Sensory symptoms (pain, paresthesia, Lhermitte's sign)	140	18 (46.2)	62 (41.1)	40 (44.0)	20 (35.1)	0.471
Facial motor symptoms	11	1 (2.6)	4 (2.6)	3 (3.3)	3 (5.3)	0.398
Facial sensory symptoms	19	3 (7.7)	8 (5.3)	5 (5.5)	3 (5.3)	0.737
Oculomotor impairment	68	7 (17.9)	33 (21.9)	15 (16.5)	13 (22.8)	0.975
Vertigo, hypoacousia	43	4 (10.3)	20 (13.2)	9 (9.9)	10 (17.5)	0.575
Speech/swallowing impairment	5	0 (0)	3 (2.0)	1 (1.1)	1 (1.8)	0.831
Mental deterioration	4	0 (0)	1 (.7)	2 (2.2)	1 (1.8)	0.231
Psychiatric symptoms	5	0 (0)	3 (2.0)	1 (1.1)	1 (1.8)	0.831
Paroxysmal symptoms	6	1 (2.6)	1 (.7)	2 (2.2)	2 (3.5)	0.347

* Based on Mann-Whitney test

Complete recovery after the first attack, longer interval between the first and second attacks, fewer symptoms at presentation, shorter disease duration, and relapsing-remitting course were associated with less disability and better prognosis (Tables 4 and 5).

**Table 4:** Associations of disease course and attack properties with disease severity

		Total	Chronic	Mild-moderate	Advanced-accelerated	Aggressive-malignant	P*
Recovery	Complete	236	37 (94.9)	128 (88.3)	48 (60.8)	23 (42.6)	< 0.001
	Incomplete	46	2 (5.1)	12 (8.3)	22 (27.8)	10 (18.5)	
	No recovery	35	0 (0)	5 (3.4)	9 (11.4)	21 (38.9)	
Progression	Relapsing-remitting	222	39 (100.0)	139 (92.1)	38 (42.2)	6 (10.5)	< 0.001
	Secondary progressive	71	0 (0)	6 (4.0)	38 (42.2)	27 (47.4)	
	Primary progressive	33	0 (0)	4 (2.6)	9 (10.0)	20 (35.1)	
	Progressive-remitting	11	0 (0)	2 (1.3)	5 (5.6)	4 (7.0)	

* Based on Kruskal-Wallis test

**Table 5:** Associations of disease severity and age, disease duration, attack intervals, and number of presenting symptoms

	Total		Chronic		Mild-moderate		Advanced - Accelerated		Aggressive - Malignant		P

	Mean ± SD	Median (Range)	Mean ± SD	Median (Range)	Mean ± SD	Median (Range)	Mean ± SD	Median (Range)	Mean ± SD	Median (Range)	
Age (years)	34.0 ± 10.0	33.0 (12-58)	30.0 ± 8.0	30 (15-46)	33.0 ± 10.0	32 (12-55)	35.0 ± 10.0	34 (17-58)	37.0 ± 10.0	37 (16-58)	< 0.001
Disease duration (years)	7.6 ± 5.6	6.0 (1-30)	6.2 ± 3.5	5 (3-16)	7.4 ± 6.2	5 (1-30)	8.5 ± 5.4	8 (1-25)	7.7 ± 5.2	6 (1-29)	0.022
Age at disease onset (years)	26.4 ± 8.7	24.5(5-54)	24.0 ± 6.9	23 (11-43)	25.8 ± 8.8	24 (10-54)	26.7 ± 8.5	24 (5-47)	29.0 ± 9.4	27 (14-51)	< 0.001
Number of symptoms	2.0 ± 1.5	1.5(0-11)	1.5 ± 0.9	1(0-4)	1.9 ± 1.3	1 (0-8)	2.2 ± 1.6	2 (0-8)	2.6 ± 1.9	2 (1-11)	< 0.001
Interval between the 1^st^ and 2^nd^ attacks (Months)	26.0 ± 35.0	12.0 (0-204)	30.0 ± 25.0	24 (3-132)	34.0 ± 40.0	12(0-204)	21.0 ± 33.0	12 (0-180)	11.0 ± 20.0	3 (0-108)	< 0.001

### Results of multivariate analysis

Ordinal logistic regression showed more severe disease to be related with increasing age of onset, higher number of presenting symptoms, having polysymptomatic disease onset, difficulty in walking, upper and lower extremity dysfunction, and progressive disease course. However, when multiple logistic regression was performed, the strongest determinant of disease severity was disease course (OR = 49.12 for secondary progressive course and OR = 53.25 for primary progressive ± relapse course). Difficulty in walking had a borderline association with disease severity OR = 2.31; P = 0.055). Although increasing number of symptoms at onset was found to be associated with more severe disease, the relation was not statistically significant (Table 6).

**Table 6:** Comparison of univariate and multivariate analysis

Parameter	Univariate*			Multivariate**		

	OR	95% CI	P	OR	95% CI	P
Gender			0.260			
Female	1(Ref)	-		1(Ref)	-	
Male	1.32	0.81-2.16		1.00	0.49-2.04	0.994
Age at disease onset (years)	1.03	1.01-1.06	0.004	0.96	0.93-1.00	0.042
Disease duration	1.03	0.99-1.07	0.088	0.93	0.88-0.99	0.017
Education (years)			< 0.001			0.074
> 16	1 (Ref)	-		1 (Ref)	-	
12-16	0.89	0.34-2.34		0.46	0.15-1.40	
5-12	1.88	0.73-4.82		0.71	0.23-2.16	
< 5	6.02	1.81-20.1		2.21	0.47-10.34	
Positive family history			0.805			
No	1 (Ref)	-				
Yes	1.08	0.59-1.96		0.87	0.42-1.80	0.700
Disease course				< 0.001		< 0.001
RR	1 (Ref)	-				
SP	28.81	15.08-55.02		49.14	16.14-149.62	
PP + PR	44.49	20.56-94.51		53.25	21.26-133.37	
Number of symptoms	1.31	1.14-1.49	< 0.001	1.03	0.73-1.46	0.852
Polysymptomatic onset						
No	1 (Ref)	-				
Yes	2.24	1.49-3.33	< 0.001	-	-	
Presenting symptoms						
Difficulty in walking	3.72	2.37-5.83	< 0.001	2.31	0.98-5.42	0.055
Lower extremity dysfunction	3.74	2.30-6.10	< 0.001	1.91	0.73-4.99	0.189
Upper extremity dysfunction	1.85	1.05-3.24	0.033	0.66	0.26-1.68	0.388
Optic neuritis	0.64	0.42-0.96	0.033	0.61	0.27-1.36	0.225
Bladder/bowel dysfunction	1.68	0.76-3.7	0.196	1.62	0.51-5.16	0.417
Sensory symptoms	0.86	0.58-1.29	0.471	0.73	0.35-1.52	0.397
Oculomotor impairment	0.99	0.61-1.62	0.975	0.80	0.34-1.87	0.605
Vertigo, hypoacousia	1.19	0.65-2.16	0.569	0.97	0.37-2.57	0.951

OR: Odds ratio for getting worse conditions

* Based on ordinal logistic regression

** Based on multiple ordinal logistic regression

## Discussion

Comparison of the results of the many studies designed to determine prognostic factors in MS shows different findings and inconsistency about demographic and clinical prognostic determinants (Table 7). Possible explanations for such discrepancies observed in these studies are as follows:

1. Some of these studies are population-based while others are clinic-based (e.g. the current study in Tehran). Clinic-based studies may contain patients with more medical interventions. However, many chronic patients may never seek medical care. In referral centers (like those in the present study), on the other hand, one may find more patients with aggressive disease.
2. Different diagnostic criteria for patient inclusion (definite or possible MS) can also be a cause of discrepancy.
3. Some of the mentioned studies are prospective while others have a retrospective design. Prospective data collection potentially brings increased accuracy unless patient assessments are very infrequent or the desired outcome is reached in between these sparse examinations. In retrospective assignment, there are fewer excluded patients and thus less certainty. Our study had the advantage of using MSSS to rate disability. Therefore, it had the potential of determining disease severity according to one assessment in a cross sectional study.
4. Another source of variable results in different studies is different definitions used for chronic cases. The mostly used definition is EDSS ≤ 2 or 3 after 10 years. In this definition, we lose some patients every decade because of the progressive nature of the disease. By using MSSS and severity subgroups in the current study, we insisted that every case of MS is progressive but the rate of progress is different for each patient. With this idea, one can understand that chronic MS is not a static course and has very slow progression of disability over time.
5. Some aspects of disability of patients with MS, such as fatigue, cognitive problems, and upper extremity dysfunction, are not considered in EDSS. Hence, a chronic case according to EDSS may have many problems not considered in the scale and may not be truly a chronic case.

**Table 7:** Clinical and demographic features associated with disability and secondary progression in different natural history studies^8^

Location	Endpoint	From onset of multiple sclerosis		Age at the onset of the disability	

		Positively associated with better outcome	Not associated	Positively associated with better outcome	Not associated
Göteborg,Sweden	DSS 6	Younger onset age; monoregional onset symptoms; RR disease course	Gender; specific onset symptoms: optic neuritis, brainstem,spinal symptoms;season of birth	--	--
London, Ontario,Canada	DSS 6	Female gender;younger onset age; RR disease course; onset symptoms: presence of optic nerve involvement; absence of motor (insidious) or limb ataxia/balance symptoms	Onset symptoms: sensory, motor (acute), diplopia, and/or vertigo	--	--
Lyon, France	EDMUS impairment scale (DSS adapted)	Female gender; younger onset age; onset symptoms: presence of optic neuritis; absence of long-tract involvement; RR disease course	Brainstem involvement	Female gender; older onset age; RR disease course (for DSS 4&6, not7)	Onset symptoms: optic neuritis; brainstem or long tracts involvement
Lyon, France	SPMS	Female gender	Onset symptoms: long tracts, optic neuritis, brainstem	--	--
British Columbia, Canada	DSS 6	Female gender; younger onset age; RR disease course	Onset symptoms: motor, sensory, optic neuropathy, or cerebellar, ataxia, or brainstem; month or season of birth	Older onset age; RR diseasecourse	Gender; onset symptoms: motor, sensory,optic neuropathy, or cerebellar, ataxia, or brainstem; month or season of birth

DSS: Disability Status Scale; EDSS: Expanded Disability Status Scale; EDMUS: European Database for Multiple Sclerosis; NA: Not available; RR: Relapsing-remitting; SPMS: Secondary-progressive multiple sclerosis

Studies have been placed in approximately chronological order, starting with the oldest. Findings from multivariate analysis were reported where possible.

### Gender and disease severity

The influence of sex on disease frequency and long-term prognosis has been assessed in numerous studies. We know that MS affects women more than men with the ratio of 3.2 in all types of MS. In primary progressive MS, this ratio is near one with a small male predominance. The female to male ratio has been reported as 2.6, 3.1, 3.4, and 4.5 in some Iranian studies.^9–12^ We found a ratio of 3.69 in our study. Moreover, in recent years, the incidence of the disease has had a higher increase in women than in men.^13^ This difference might be due to the increased availability of medical care for women, lifestyle modifications, and also a bias of remembrance and report of disease state (men rarely remember accurate time of health related events but women remember precisely when they had any symptom and women seek help earlier) which make female patients report and remember the health events with more details.^14^ Nevertheless, the mentioned reasons cannot explain this much difference between the two sexes. We also evaluated the effects of gender on disease severity but failed to find any significant between the two genders. While multiple natural history studies have found female gender as a better prognostic indicator,^8^ others have not (Table 7).

More importantly, multivariate analysis showed that sex did not have a strong influence on the long-term prognosis when other factors were taken into account.^3^ A previous study found female sex as a risk factor for progression of clinically isolated syndrome (CIS) to MS.^3^ However, other studies on CIS which were not limited to ocular involvement did not report gender to affect the outcome.^3^

The influence of sex on disease severity is still under investigation. Recent studies have not found any differences between the two sexes in the severity of axonal damage, mature oligodendrocytes count, or oligodendrocytes loss in early MS lesions. Pregnant women with MS experience a 70% reduction in relapses during pregnancy and a rise in attacks in the first trimester. The disease reaches its level before pregnancy six months after delivery.^15^ Treatment of relapsing-remitting MS with estriol, a candidate for protective effect against attacks during pregnancy, reduced number of inflammatory lesions in magnetic resonance imaging (MRI). However, such a reduction was not observed secondary progressive MS.^15^ Calcitriol, another candidate for protective effects during pregnancy, peaks in the third trimester and decreases after delivery.^15^ In an experiment on mice with experimental autoimmune encephalomyelitis (EAE), calcitriol had protective effects only in mice with ovaries, a witness for importance of estrogens (not only estriol) in protection.^15^ In addition, some studies have shown decreased frequency of attacks during exclusive breast feeding.^15^

In general, neither descriptive nor pathophysiological studies have agreed on better prognosis in female population. Hormones seem to only affect attack frequency and not long-term disability.

### Age at disease onset and disease severity

According to the current study and previous research, younger age at disease onset is associated with more favorable outcome. This seems to be related mostly with better recovery and central nervous system repair in younger patients.

### Disease course and severity

It is evident from many natural history studies that relapsing onset disease has more favorable prognosis in contrast to progressive disease. In our study and in univariate analysis, secondary progressive course was 29 times more susceptible to severe disease than relapsing-remitting course. This value was 44 for primary progressive and primary relapsing courses. When a multiple logistic regression was performed, the strongest predictor of disease severity was disease course.

A recent study found that 9% of patients with primary progressive MS had a chronic course (EDSS < 3 in 10 years).^16^ Although primary progressive MS has an insidious onset, a falsely shorter duration is considered for the disease. This may be a possible explanation why the disease is usually considered to rapidly progress.

In 1990, four pathologic patterns were described in MS lesions. According to them, some lesions appear to be chiefly inflammatory (types I and II) with retention of active oligodendrocytes derived from identifiable precursor cells and evidence of remyelination. The most common pathological pattern seen (type II) had inflammatory infiltrates and deposition of complement and immunoglobulin G. In other patients, extensive destruction of oligodendrocytes, little replacement, and closer resemblance to a viral or toxic cell apoptosis or necrosis was found (types III and IV). Type IV was the rarest condition (a real oligodendroglioma) and was only observed in patients with primary progressive disease. The most common pattern in the mentioned patients was type II changes.^17^ These findings may support the idea that different disease courses distinguished in MS may be a reflection of different neuropathological mechanisms. In other words, different pathological types may be different disease conditions now all known as primary progressive MS.

The current study emphasized the impossibility of predicting disease severity and rate of disability progression according to early clinical and demographic factors. Therefore, it is not possible to predict a chronic course early in the disease. Other factors which may be a combination of demographic, clinical, and image findings and biomarkers should be investigated for the purpose of long-term disability prediction.

We could not find any explanation for the effects of education on disease severity observed in our data.
